# Cinnamon and Its Metabolite Sodium Benzoate Attenuate the Activation of p21^rac^ and Protect Memory and Learning in an Animal Model of Alzheimer’s Disease

**DOI:** 10.1371/journal.pone.0130398

**Published:** 2015-06-23

**Authors:** Khushbu K. Modi, Avik Roy, Saurabh Brahmachari, Suresh B. Rangasamy, Kalipada Pahan

**Affiliations:** 1 Department of Neurological Sciences, Rush University Medical Center, Chicago, United States of America; 2 Division of Research and Development, Jesse Brown Veterans Affairs Medical Center, 820 South Damen Avenue, Chicago, United States of America; School of Medicine and Health Sciences, University of North Dakota, UNITED STATES

## Abstract

This study underlines the importance of cinnamon, a commonly used natural spice and flavoring material, and its metabolite sodium benzoate (NaB) in attenuating oxidative stress and protecting memory and learning in an animal model of Alzheimer’s disease (AD). NaB, but not sodium formate, was found to inhibit LPS-induced production of reactive oxygen species (ROS) in mouse microglial cells. Similarly, NaB also inhibited fibrillar amyloid beta (Aβ)- and 1-methyl-4-phenylpyridinium(+)-induced microglial production of ROS. Although NaB reduced the level of cholesterol *in vivo* in mice, reversal of the inhibitory effect of NaB on ROS production by mevalonate, and geranylgeranyl pyrophosphate, but not cholesterol, suggests that depletion of intermediates, but not end products, of the mevalonate pathway is involved in the antioxidant effect of NaB. Furthermore, we demonstrate that an inhibitor of p21^rac^ geranylgeranyl protein transferase suppressed the production of ROS and that NaB suppressed the activation of p21^rac^ in microglia. As expected, marked activation of p21^rac^ was observed in the hippocampus of subjects with AD and 5XFAD transgenic (Tg) mouse model of AD. However, oral feeding of cinnamon (*Cinnamonum verum*) powder and NaB suppressed the activation of p21^rac^ and attenuated oxidative stress in the hippocampus of Tg mice as evident by decreased dihydroethidium (DHE) and nitrotyrosine staining, reduced homocysteine level and increased level of reduced glutathione. This was accompanied by suppression of neuronal apoptosis, inhibition of glial activation, and reduction of Aβ burden in the hippocampus and protection of memory and learning in transgenic mice. Therefore, cinnamon powder may be a promising natural supplement in halting or delaying the progression of AD.

## Introduction

Alzheimer's disease (AD) is the most common human neurodegenerative disorder and the main cause of dementia among the elderly. Since the elderly population of the world is increasing, the prevalence of AD is also on the rise worldwide. The majority of AD cases (90–95%) are sporadic and the remainder familial. However, neuropathologically, in both cases, the disease is characterized by two hallmark lesions: neurofibrillary tangles and neuritic plaques. Neuritic plaques are composed of aggregates of Aβ protein, a 40–43 amino acid proteolytic fragment derived from the amyloid precursor protein that is over-expressed in AD while NFTs are composed of hyperphosphorylated microtubule-associated protein tau [1,2]. Although the etiology of sporadic AD is poorly understood, numerous studies suggest an important role of oxidative stress in the pathogenesis of AD [3]. It has been shown that the levels of protein carbonyls and 3-nitrotyrosine as well as markers of oxidative damage to nucleic acids, such as 8-hydroxydeoxyguanosine and 8-hydroxyguanosine, are elevated in AD brains [4,5]. Similarly, malondialdehyde and 4-hydroxynonenal, products of lipid peroxidation, are also increased in multiple brain regions of patients with AD or mild cognitive impairment (MCI) [6]. Furthermore, oxidative stress is known stimulate the production of Aβ1-40/42 in the CNS of AD patients via increasing the activation of β- and γ-secretase and decreasing the activity of α-secretase [3,7]. Therefore, attenuation of oxidative stress in the CNS of AD patients is an important area of research.

Although there are other antioxidants, here, we introduce a natural approach to suppress oxidative stress in the hippocampus. Cinnamon is a commonly used natural spice and flavoring material used for centuries throughout the world. Here, we delineated that oral administration of cinnamon powder to 5XFAD transgenic mice produced NaB in the hippocampus. Interestingly, NaB decreased the production of reactive oxygen species (ROS) in activated microglia via suppressing the activation of p21^rac^, a member of the NADPH oxidase complex. Furthermore, oral administration of both cinnamon and NaB inhibited the activation of p21^rac^ and reduced oxidative stress in the hippocampus of 5XFAD mice. Treatment of 5XFAD mice with cinnamon and NaB was also associated with suppression of neuronal apoptosis and reduction of Aβ burden in the hippocampus and protection of memory and learning in 5XFAD mice. These results suggest that cinnamon may be used to control oxidative stress-associated neurodegeneration in AD patients via attenuation of p21^rac^ activation.

## Materials and Methods

Animal maintaining and experiments were in accordance with National Institute of Health guidelines and were approved by the Institutional Animal Care and Use committee of the Rush University of Medical Center, Chicago, IL. Whenever needed, animals were anesthetized by ketamine/xylazine injectable.

### Reagents

Fetal bovine serum (FBS) and DMEM/F-12 were from Mediatech (Washington, DC). Sodium benzoate (NaB), sodium formate (NaFO) and 1-methyl-4-phenylpyridinium (MPP^+^) were purchased from Sigma Aldrich (St. Louis, MO). Original Ceylon cinnamon (*Cinnamonum verum*) in ground form was obtained from Indus Organics (San Ramon, CA). Amyloid β peptide (1–42) was obtained from Bachem Bioscience. Rat anti-mouse Iba1 was purchased from Chemicon. Aβ (N) 82E1 monoclonal antibodies were received from IBL America (Minneapolis, MN). Anti phospho-Tau monoclonal antibody (clone PHF13) was purchased from Millipore (catalog # 05–885). Anti-tau monoclonal antibody (TAU-5) that detects total tau was purchased from Calbiochem (catalog # 577801). Alexa-fluor antibodies used in immunostaining were obtained from Jackson ImmunoResearch and IR-dye-labeled reagents used for immunoblotting were from Li-Cor Biosciences.

### Human brain tissues

Autopsy brain tissues from two male and two female AD patients and four control subjects with no-cognitive impairment (NCI) were obtained from the University of Iowa Deeded Body Program. AD patients and control subjects did not differ significantly for their mean age at death (AD, 80.25 ± 3.4 years; NCI, 76.5 ± 5.2 years). The mean postmortem interval for AD and NCI were 5.2 ± 0.96 and 4.2 ± 0.63 h, respectively.

### Preparation of Fibrillar Aβ

Fibrillar Aβ1–42 and control reverse peptide Aβ42–1 (Bachem Bioscience) were prepared by incubating freshly solubilized peptides at 50 μM in sterile distilled water at 37°C for 5 days [8,9].

### Animals and cinnamon treatment

B6SJL-Tg(APPSwFlLon,PSEN1*M146L*L286V) 6799Vas/J transgenic (5XFAD) mice were purchased from Jackson Laboratories (Bar Harbor, ME). Six month old male 5XFAD mice were treated with cinnamon (100 mg/kg body wt/d) via gavage for 2 months followed by monitoring memory and learning and hippocampal histochemical and biochemical assays. Briefly, cinnamon (*Cinnamonum verum*) powder was mixed in 0.5% methylcellulose (MC) and 5XFAD mice were gavaged 100 μL cinnamon-mixed MC once daily using gavage needle as described [10]. Therefore, control 5XFAD mice received only MC as vehicle.

### Monitoring microglial ROS production

Mouse BV-2 microglial cells (kind gift from Virginia Bocchini, University of Perugia) were maintained in Dulbecco's modified Eagle's medium/F-12 containing 10% (v/v) fetal bovine serum. These cells, cultured in 8-well chamber slides, were treated with LPS, fibrillar Aβ1–42 or MPP^+^ under serum-free condition. At different time points of stimulation, supernatants were removed and cells were washed with Hank’s buffered salt solution (HBSS) followed by addition of 100 μl of 25 μM carboxy-H_2_DCFDA to each well for 30 min of incubation as described by us [11]. During the last five minutes of incubation, Hoechst 33342 was added to each well at a dilution of 1:1000 for staining nuclei. Cells were then washed with HBSS, mounted with DPX mounting media and observed under an Olympus IX81 fluorescent microscope.

### Superoxide measurement

Superoxide production was detected by LumiMax Superoxide Anion Detection Kit (Stratagene) as described by us [12].

### Activation of p21^rac^

The activated p21^*rac*^ interacts with p21-activated kinase (PAK). Accordingly, p21^*rac*^-interacting domain (RID) of PAK binds specifically to the GTP-bound (active) form of p21^*rac*^. Therefore, using an assay kit from Upstate Biotechnology (Waltham, MA), microglial cells were homogenized with lysis buffer containing inhibitors of different proteases and kinases followed by immuno-pull down of active p21^*rac*^ using PAK-RID-GST beads. Then the amount of activated p21^*rac*^ was determined in GST beads by a Western blot using a p21^*rac*^ specific antibody.

### Barnes Maze

Barnes maze was performed as described by us [13,14,15]. Briefly, for Barnes maze, mice were trained for 2 consecutive days followed by examination on day 3. During training, the overnight food-deprived mouse was placed in the middle of the maze in a 10 cm high cylindrical black start chamber. After 10 s, the start chamber was removed to allow the mouse to move around the maze to find out the color food chips in the baited tunnel. The session was ended when the mouse entered the baited tunnel. The tunnel was always located underneath the same hole (stable within the spatial environment), which was randomly determined for each mouse. After each training session, maze and escape tunnel were thoroughly cleaned with a mild detergent to avoid instinctive odor avoidance due to mouse's odor from the familiar object. On day 3, the maze was illuminated with high wattage light that generated enough light and heat to motivate animals to enter into the escape tunnel [16], allowing us to measure latency (duration before all four paws were on the floor of the escape box) and errors (incorrect responses before all four paws were on the floor of the escape box).

### T Maze

T maze experiments were performed as described by us [13,15]. Briefly, mice were habituated in the T-maze for two days under food-deprived conditions so that animals can eat food rewards at least five times during 10 minutes period of training. During each trial, mice were placed in the start point for 30 s and then forced to make a right arm turn which was always baited with color food chips. On entering the right arm, they were allowed to stay there for 30–45 s, then returned to the start point, held for 30 s and then allowed to make right turn again. As described above, after each training session, T maze was thoroughly cleaned with a mild detergent. On day 3, mice were tested for making positive turns and negative turns. The reward side is always associated with a visual cue. Number of times the animal eats the food reward would be considered as a positive turn.

### Novel Object Recognition Task

Novel object recognition task was performed to monitor the short term memory as described by others [17] and us [15]. Briefly, during training, mice were placed in a square novel box (20 inches long by 8 inches high) surrounded with infrared sensor. Two plastic toys (between 2.5 and 3 inches) that varied in color, shape, and texture were placed in specific locations in the environment 18 inches away from each other. The mice were able to explore freely the environment and objects for 15 min and then were placed back into their individual home cages. After 30 mins, mice were placed back into the environment with two objects in the same locations, but now one of the familiar objects was replaced with a third novel object. The mice were then again allowed to explore freely both objects for 15 min. The objects were thoroughly cleaned with a mild detergent.

### Immunoblotting

Western blotting was performed as described earlier [18,19,20] with modifications. Briefly, cells were scraped in lysis buffer, transferred to microfuge tubes and spun into pellet. The supernatant was collected and analyzed for protein concentration via the Bradford method (Bio-Rad). SDS sample buffer was added to 30–50 μg total protein and the sample was boiled for 5 min. Denatured samples were electrophoresed on NuPAGE Novex 4–12% Bis-Tris gels (Invitrogen) and proteins transferred onto a nitrocellulose membrane (Bio-Rad) using the Thermo-Pierce Fast Semi-Dry Blotter. The membrane was then washed for 15 min in TBS plus Tween 20 (TBST) and blocked for 1 h in TBST containing BSA. Next, membranes were incubated overnight at 4°C under shaking conditions with primary antibody. The next day, membranes were washed in TBST for 1 h, incubated with secondary antibody (Li-Cor Biosciences) for 1 h at room temperature, washed for one more hour and visualized under the Odyssey Infrared Imaging System (Li-COR, Lincoln, NE).

### ELISA

For Aβ1–42 ELISA, hippocampal tissues were homogenized in TBS, pelleted for 30 min x 150,000*g*. The pellet was resuspended in 3 volumes (wt/vol original tissue weight) of TBS+1% Triton X-100, pelleted for 30 min x 150,000*g* and the supernatant recovered and stored. Samples were assayed for protein concentration and diluted 10 fold prior to performing ELISA according to manufacturer’s instruction (BioLegend, SIG-38956).

### Immunostaining

Brains were kept in 4% paraformaldehyde and 30-μm slices were sectioned in a cryostat followed by immunostaining as described before [21,22]. Briefly, the samples were blocked in 3% bovine serum albumin in PBS for 1 hour. Then brain sections were incubated with permeabilization reagent containing 0.05% Tween 20 and 0.25% Triton X-100 in PBS for 5 min followed by double-labelling with two antibodies. The samples were then washed four times 10 min each in PBST followed by incubation with secondary antibodies conjugated to fluorophores with non-overlapping emission spectra with Alexa Fluor 488 and Alexa Fluor 647 (Jackson ImmunoResearch Laboratories, Inc.). After 1 h incubation at room temperature, the samples were washed in PBST, mounted and observed under a Bio-Rad (Hercules, CA) MRC1024ES confocal laser-scanning microscope.

### TUNEL and NeuN double-labeling

This was performed as described by us earlier [13]. Briefly, tissue samples were blocked using blocking buffer followed by treatment with 20 μg/ml proteinase K for 15 min at room temperature and one wash with PBS. Next, the samples were incubated for 90 min in terminal deoxynucleotidyl transferase (TdT) equilibration buffer containing anti-NeuN antibody. After three washes in PBST, the sections were incubated in fluorescein-fragEL TdT reaction mix containing TdT enzyme and secondary antibody for 60 min at 37°C. Prior to mounting, the samples were washed twice in PBS. Finally, the samples were mounted using mounting media containing 4,6,-DiAmidino-2-PhenylIndole (DAPI), which allows the visualization of total cell population and observed for fluorescein-labeled DNA fragments.

### Statistical Analysis

All values were expressed as means ± SD of three independent experiments. Statistical differences between means were calculated by the Student's *t*-test. A *p-* value of less than 0.05 (p<0.05) was considered statistically significant. Differences in behavioral measures were examined by independent one-way ANOVA using SPSS. Homogeneity of variance between test groups was examined using Levene's test. *Post-hoc* analyses were conducted using Tukey’s or Games-Howell tests, where appropriate. *p* < 0.05 was considered statistically significant.

## Results

### Oral administration of cinnamon (Cinnamonum verum) powder increased the level of sodium benzoate (NaB) in the hippocampus of 5XFAD transgenic (Tg) mice

**Fig 1:**
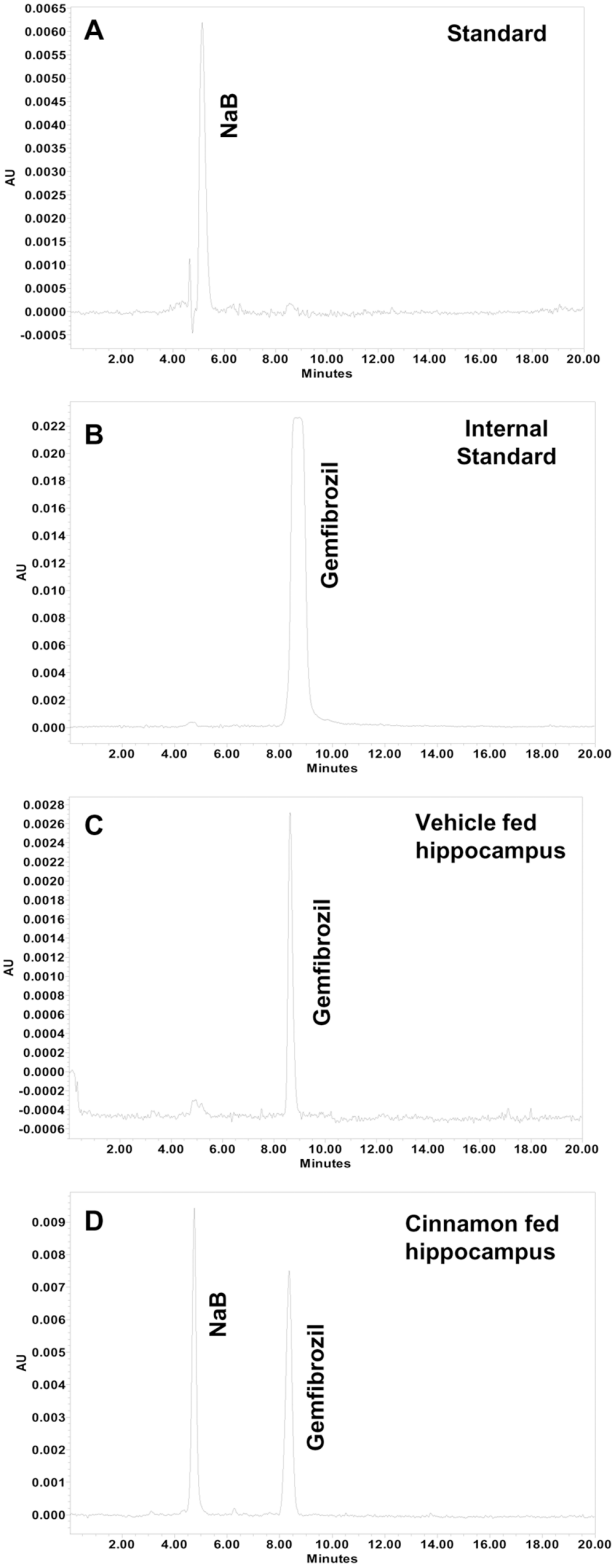
Oral administration of cinnamon powder (*Cinnamonum verum*) produces sodium benzoate (NaB) *in vivo* in the hippocampus of 5XFAD Tg mice. Six-month old Tg mice (n = 3) were treated with cinnamon powder (100 mg/kg body wt/d) in 100 μl 0.5% methylcellulose as vehicle orally. After seven days of treatment, the level of NaB was monitored in the hippocampus by HPLC (A, NaB standard; B, Gemfibrozil internal standard; C, hippocampus of vehicle-fed mice; D, hippocampus of cinnamon-fed mice). Briefly, mice were sacrificed and 25 mg hippocampal tissue was collected in 1 ml chloroform: methanol (2:1) extraction solvent containing 0.05M perchloric acid. Gemfibrozil was added as an internal standard. Tissues were homogenized and centrifuged at a speed of 20,000g for 10 mins. Organic and aqueous phases were separated carefully and 10 μl aqueous phase was analyzed for the detection of NaB in Waters 2695 separation module HPLC system with the help of “Em-power pro” software and Phenomenex Luna 5μ C18 100A column (250 x 4.6 mm; 280-nm UV wavelength) using acetonitrile as mobile phase at the flow rate of 0.2 ml/min.

Two major types of cinnamon that are available in the US are Chinese cinnamon (*Cinnamonum cassia*) and original Ceylon cinnamon (*Cinnamonum verum* or *Cinnamonum zylencum*). Recently by mass spectrometric analysis, we have found that *Cinnamonum verum* is much more pure than *Cinnamonum cassia* [18]. Although both *Cinnamonum cassia* and *Cinnamonum verum* contain cinnamaldehyde as the major peak, *Cinnamonum cassia* contains more styrene, benzene, 1,1’-(2-butene-1,4-diyl)bis-, benzene, 1,1’-(1,2-cyclobutanediyl)bis-, palmitic acid, stearic acid, 4-phenylbutyl chloride, and (2,3-diphenylcyclopropyl)methyl phenyl sulfoxide than *Cinnamonum verum* [18]. Most importantly, *Cinnamonum cassia*, but not *Cinnamonum verum*, contains small amount of toxic 1-benzopyran-2-one or coumarin [18]. Therefore, here, we used *Cinnamonum verum* throughout this study. At first, we examined if oral administration of ground *Cinnamonum verum* in 5XFAD transgenic (Tg) mice increased the level of NaB in the hippocampus. After 7 d of oral administration of *Cinnamonum verum* powder at a dose 100 mg/kg body wt/d, a sharp peak for NaB was detected in the hippocampus of 5XFAD Tg mice (Fig 1). On the other hand, we did not find any peak for NaB in the hippocampus of vehicle (methyl cellulose)-fed Tg mice (Fig 1).

### NaB inhibits the production of reactive oxygen species (ROS) from activated microglia

**Fig 2:**
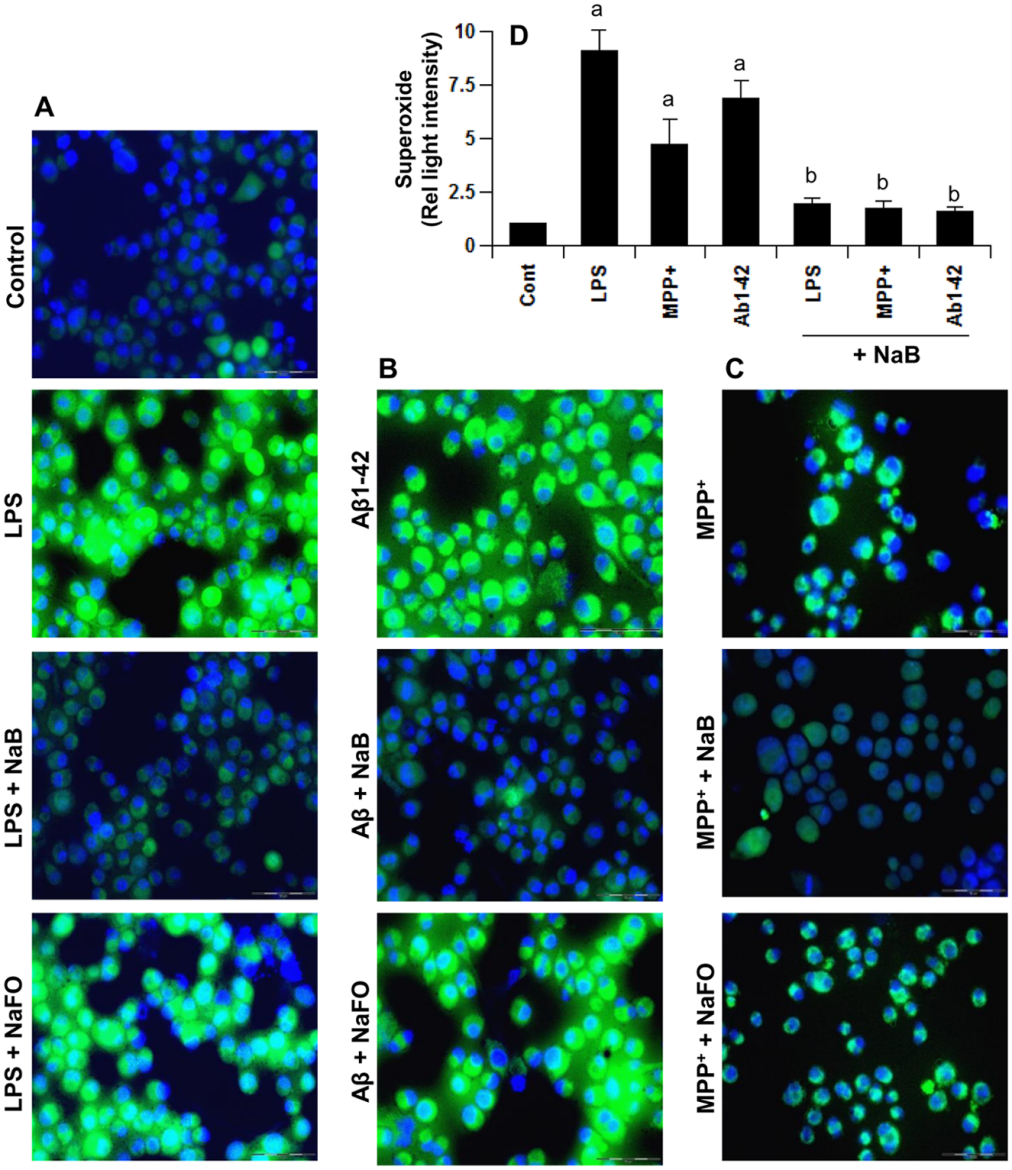
Cinnamon metabolite sodium benzoate (NaB) reduces the production of reactive oxygen species (ROS) in activated microglia. BV-2 microglial cells pretreated with 500 μM NaB for 6 h were stimulated with 1 μg/ml LPS (A), 1 μM fibrillar Aβ1–42 (B) or 1 μM MPP^+^ (C) for 15 min followed by monitoring the generation ROS using carboxy-H_2_DCFDA. Sodium formate (NaFO) was used as a negative control of NaB. D) Under similar treatment conditions, levels of superoxide were measured in cells. Results are mean ± SD of three different experiments. ^a^p < 0.001 vs control; ^b^p < 0.001 vs stimuli.

Oxidative stress plays an important role in the pathogenesis of various neurodegenerative diseases including AD. We examined if bacterial lipopolysaccharides (LPS) induced the production of ROS from microglia and if NaB could attenuate such ROS production. To monitor the generation of intracellular ROS in BV-2 microglial cells, we used a cell-permeant fluorescent probe. As seen in Fig 2A, LPS markedly induced the generation of ROS within 15 min of stimulation. However, NaB strongly inhibited LPS-induced production of intracellular ROS (Fig 2A). This result was specific as sodium formate (NaFO) having similar chemical structure as NaB except the benzene ring had no such inhibitory effect (Fig 2A). Because various stimuli and neurotoxins are capable of producing ROS, we examined if NaB could suppress the production of ROS in response to other inflammatory stimuli. Similar to LPS, both fibrillar Aβ1–42 (an etiological reagent for AD) (Fig 2B) and MPP^+^ (a Parkinsonian toxin) (Fig 2C) also induced the production of ROS in BV-2 microglial cells within 15 min of stimulation. However, NaB, but not NaFO, inhibited (Aβ1–42)- and MPP^+^-induced ROS production in microglial cells (Fig 2B and 2C). These results suggest that cinnamon metabolite NaB could be used as an antioxidant.

**Fig 3:**
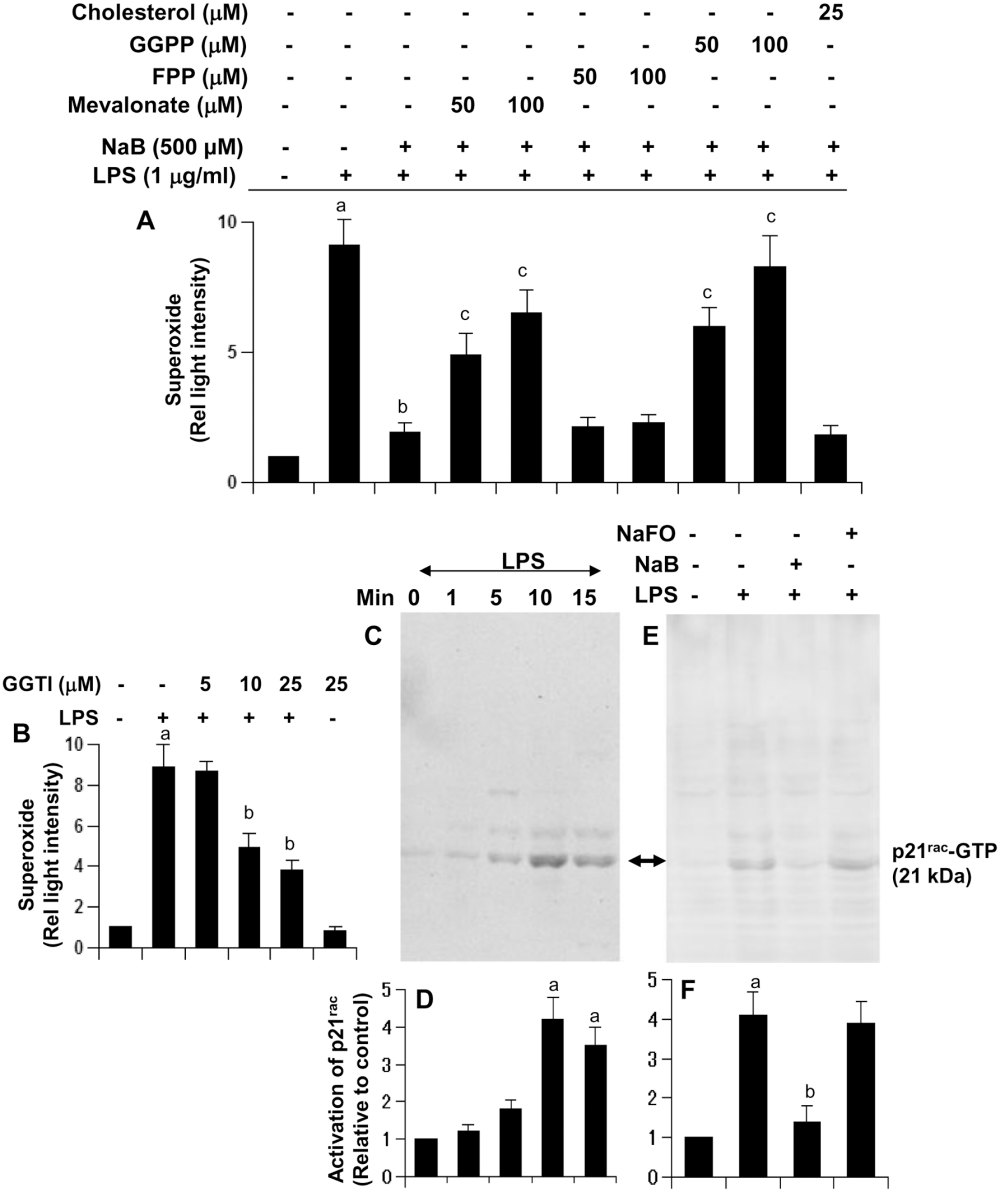
Cinnamon metabolite sodium benzoate (NaB) reduces the production of ROS and the activation of p21^rac^ in microglia. A) BV-2 microglial cells pretreated with 500 μM NaB in the presence of different intermediates of the cholesterol biosynthetic pathway for 6 h were stimulated with 1 μg/ml LPS for 15 min followed by measuring the levels of superoxide in cells. Results are mean ± SD of three different experiments. ^a^p < 0.001 vs control; ^b^p < 0.001 vs LPS; ^c^p < 0.001 vs (LPS+NaB). B) Cells pretreated with different concentrations of geranylgeranyl transferase inhibitor (GGTI) for 30 min were stimulated with LPS for 15 min followed by measuring the levels of superoxide in cells. ^a^p < 0.001 vs control; ^b^p < 0.01 vs LPS. C) Cells were stimulated with LPS under serum-free condition for different time periods followed by monitoring the activation of p21^rac^. D) Bands were scanned and presented as relative to control (0 min). ^a^p < 0.001 vs control. E) Inhibition of LPS-induced activation of p21^rac^ by NaB, but not NaFO. F) Bands were scanned and presented as relative to control. ^a^p < 0.001 vs control; ^b^p < 0.001 vs LPS.

In an earlier study, we have observed that NaB inhibits cholesterol biosynthesis and that NaB exhibits anti-inflammatory activity via modulation of mevalonate metabolites [23]. Therefore, we investigated the role of mevalonate metabolites in antioxidant effect of NaB. Interestingly, mevalonate and geranylgeranyl pyrophosphate (GGPP), but not farnesyl pyrophosphate (FPP), abrogated the inhibitory effect of NaB on the production of superoxide in microglial cells (Fig 3A). On the other hand, cholesterol had no effect on NaB-mediated inhibition of superoxide production (Fig 3A). These results suggest that depletion of intermediary products rather than end products of the mevalonate pathway is also responsible for the antioxidant effect of NaB.

### NaB suppresses the activation of p21^rac^ in mouse BV-2 microglial cells

NADPH oxidase has emerged as the most important ROS (superoxide radicals)-producing molecule in response to different inflammatory stimuli [24]. The p21^*rac*^ is an important member of activated NADPH oxidase complex, which is activated by geranylgeranylation. Accordingly, geranylgeranyl transferase inhibitor (GGTI) attenuated LPS-induced production of superoxide in BV-2 microglial cells (Fig 3B). Because GGPP reversed the antioxidant effect of NaB, NaB may inhibit the activation of p21^*rac*^. Therefore, we also examined the effect of NaB on the activation of p21^rac^ in activated microglial cells. Since activation of p21^*rac*^ is a rapid process, at different times (1, 5, 10, and 15 min) of stimulation by LPS, microglial cells were analyzed for the activation of p21^*rac*^. Although we did not see p21^*rac*^ activation at 1 min of LPS stimulation, significant activation was observed at 5 min of stimulation, and the activation was maximum at 10 min of stimulation (Fig 3C and 3D). Therefore, we examined the effect of NaB on the activation of p21^*rac*^ at 10 min of LPS stimulation. It is clearly evident from Fig 3E and 3F that NaB, but not NaFO, markedly inhibited LPS-induced activation of p21^*rac*^ in microglial cells. These results suggest that NaB attenuates the production of superoxide radicals in microglial cells probably by suppressing the activation of p21^*rac*^.

### Activation of p21^rac^ in AD

**Fig 4:**
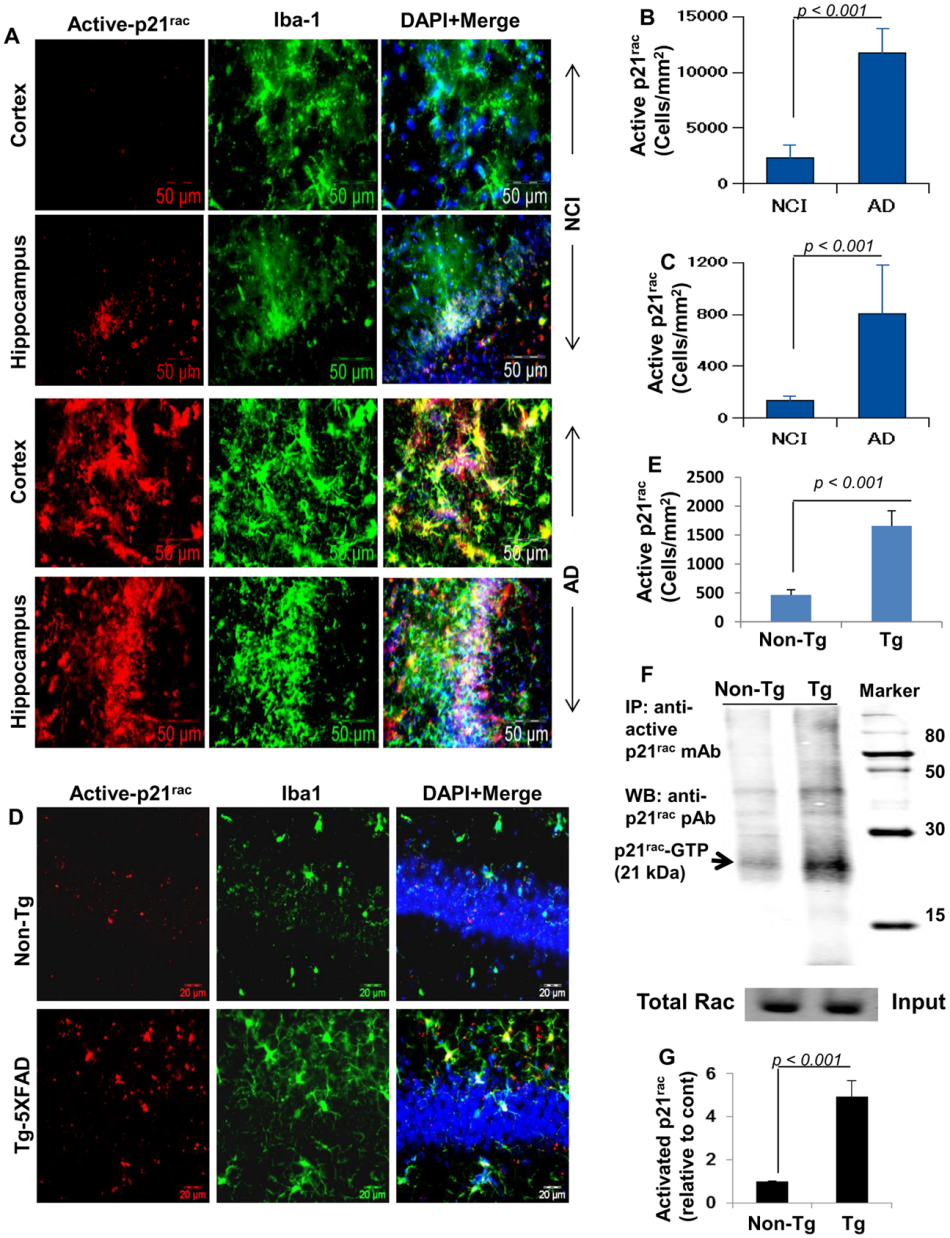
Monitoring activation of p21^rac^ *in vivo* in the CNS of AD subjects and 5XFAD transgenic mice. (A) Cortical and hippocampal sections of cases with no cognitive impairment (NCI) and AD were double-labeled with Iba-1 (microglia) and active p21^rac^ (GTP-bound p21^rac^). Results represent analysis of two sections from each of four different brains. Active p21^rac^-positive cells were counted in two sections (two images per slide) of each of four different brains per group in an Olympus IX81 fluorescence microscope using the MicroSuite imaging software (B, cortex; C, hippocampus). D) Six month old 5XFAD Tg mice and background- and age-matched non-Tg mice were perfused with PBS-paraformaldehyde followed by double-label immunofluorescence analysis of hippocampal sections for active p21^rac^ and Iba1. Results represent analysis of three sections of each of five mice per group. E) Active p21^rac^-positive cells were counted in three sections (two images per slide) of each of five different brains per group. F) To further monitor the activation of p21^rac^, hippocampal homogenates of non-Tg and Tg mice were immunoprecipitated with antibodies against active p21^rac^ followed by Western blot with antibodies against total p21^rac^. Total p21^rac^ in input was run as experimental control. G) Bands (21kDa) were scanned and presented as relative to non-Tg. Results represent analysis of four mice per group.

Activation of p21^rac^ is required for the functioning of NADPH oxidase complex and the generation of superoxide radicals [24,25]. Therefore, to understand the role of p21^rac^ in observed oxidative stress in AD, hippocampal and cortical sections of AD subjects and age-matched individuals with no cognitive impairment (NCI) were immunolabeled for activated p21^rac^ (GTP-bound p21^rac^). Since microglia is the major cell type in the CNS that produce ROS upon activation, sections were double-labeled for activated p21^rac^ and Iba-1 (microglia). The level of activated p21^rac^ was markedly higher in cortex (Fig 4A and 4B) and hippocampus (Fig 4A and 4C) of AD brain compared with NCI. We also noticed greater Iba-1 expression (microglial activation) in cortex and hippocampus of AD compared to NCI (Fig 4A). Iba-1-positive cells were also positive for activated p21^rac^ in both hippocampus and cortex of AD subjects (Fig 4A).

### Activation of p21^rac^ in 5XFAD transgenic (Tg) mice

Next, to investigate if p21^rac^ is also activated in the CNS of an animal model of AD, we examined the status of activated p21^rac^ in the hippocampus of 5XFAD (B6SJL-Tg(APPSwFlLon,PSEN1*M146L*L286V)6799Vas/J) Tg mice. Similar to that found in the CNS of AD subjects, we observed higher levels of activated p21^rac^ in the hippocampus of Tg mice as compared to age-matched non-Tg mice (Fig 4D). Number of active p21^rac^-positive cells was significantly higher in Tg mice than non-Tg mice (Fig 4E). Accordingly, we found marked increase in microglial activation as evidenced by Iba-1 immunoreactivity and many Iba-1-positive cells in the hippocampal area also co-localized with activated p21^rac^ (Fig 4D).

To further monitor the induced activity of p21^rac^, we performed immunoprecipitation-coupled Western blot analysis on hippocampal homogenates harvested from non-Tg and Tg mice. Hippocampal homogenates were immunoprecipitated with antibodies against GTP-bound p21^rac^ (activated p21^rac^) followed by Western blot analysis with antibodies against total p21^rac^. Consistent with increased immunostaining of activated p21^rac^ in the hippocampus of Tg mice, the level of active p21^rac^ protein in the hippocampus was also greater in the hippocampus of Tg mice than non-Tg mice (Fig 4F and 4G). Together, these results demonstrate the activation of p21^rac^ *in vivo* in the hippocampus of Tg mice.

### Oral administration of cinnamon powder and NaB inhibits the activation of p21^rac^ in vivo in the hippocampus of 5XFAD Tg mice

**Fig 5:**
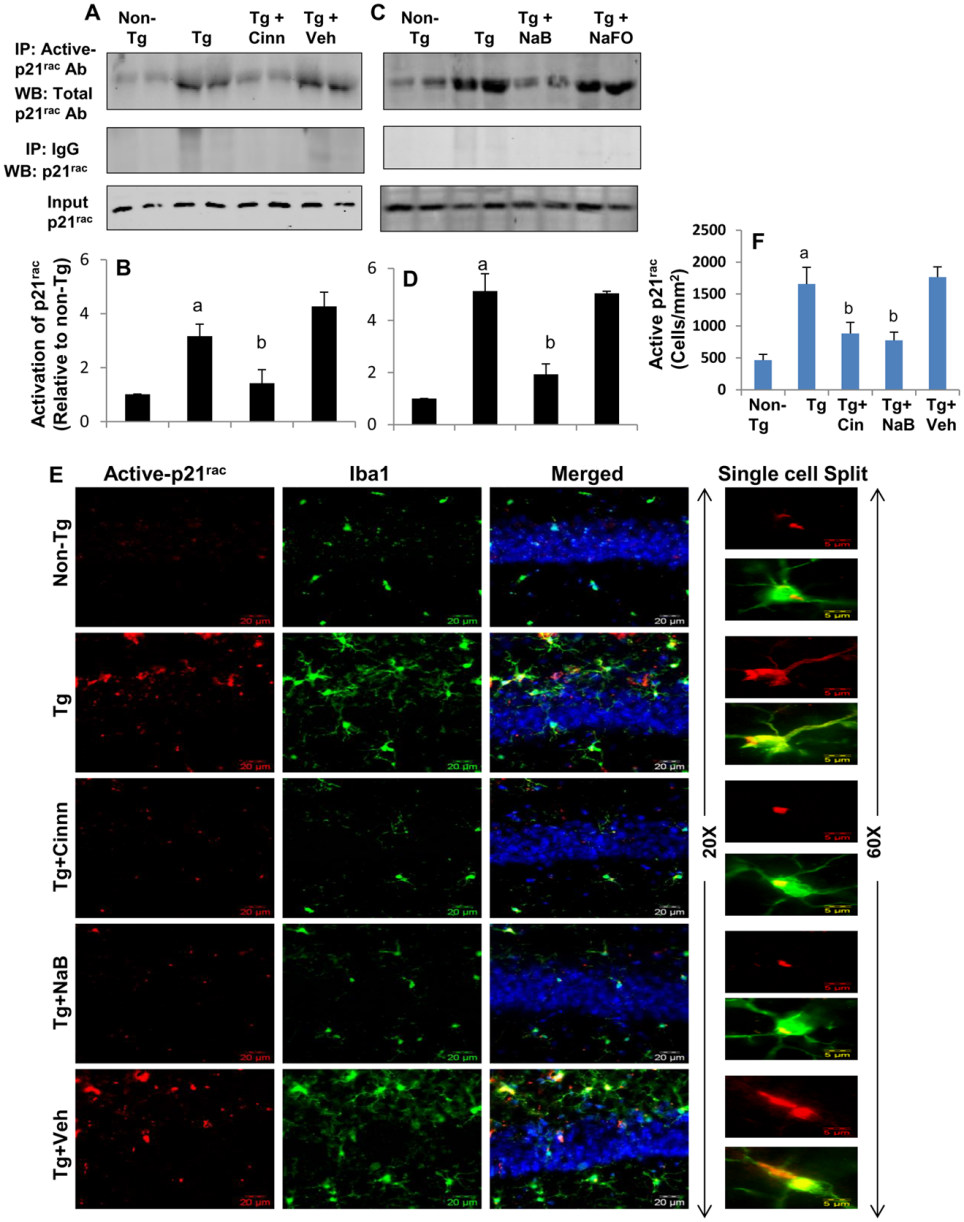
Cinnamon and its metabolite sodium benzoate (NaB) inhibit the activation of p21^rac^ in the hippocampus of 5XFAD transgenic (Tg) mice. Six-month old male Tg mice were treated with ground cinnamon (100 mg/kg body wt/d) and NaB (50 mg/kg body wt/d) orally for 60 d followed by monitoring the activation of p21^rac^ in the hippocampus (A, cinnamon; C, NaB). Bands were scanned and hippocampal p21^rac^ activation is shown in cinnamon (B) and NaB (D) treated mice. Results are mean ± SEM of six mice per group. ^a^p < 0.001 vs non-Tg; ^b^p < 0.001 vs Tg. E) Hippocampal sections were double-labeled for active p21^rac^ and Iba1 (microglia). Results represent analysis of two hippocampal sections of each of six mice per group. F) Active p21^rac^-positive cells were counted in two sections (two images per slide) of each of six different brains per group. ^a^p < 0.001 vs non-Tg; ^b^p < 0.01 vs Tg.

Because p21^rac^ is also activated in the hippocampus of AD subjects and 5XFAD Tg mice and cinnamon metabolite NaB suppressed the activation of p21^rac^ in microglial cells, we examined whether cinnamon and NaB were capable of targeting p21^rac^ *in vivo* in the hippocampus of Tg mice. As seen by immunoprecipitation-coupled Western blot analysis, oral administration of cinnamon, but not vehicle, inhibited the activation of p21^rac^ in the hippocampus of Tg mice (Fig 5A and 5B). Similarly, oral treatment of NaB, but not NaFO, also suppressed the activation of p21^rac^ (Fig 5C and 5D). These findings were confirmed by double-label immunofluorescence of hippocampal sections (Fig 5E) and counting of activated p21^rac^-positive cells (Fig 5F). As evident from Fig 5E and 5F, the level of activated p21^rac^ was inhibited in Tg mice by treatment with both cinnamon and NaB. These results suggest that oral administration of cinnamon and NaB is capable of suppressing the activation of p21^rac^ in the hippocampus of 5XFAD Tg mice.

### Oral administration of cinnamon and NaB inhibits oxidative stress and gliosis in vivo in the hippocampus of 5XFAD Tg mice

**Fig 6:**
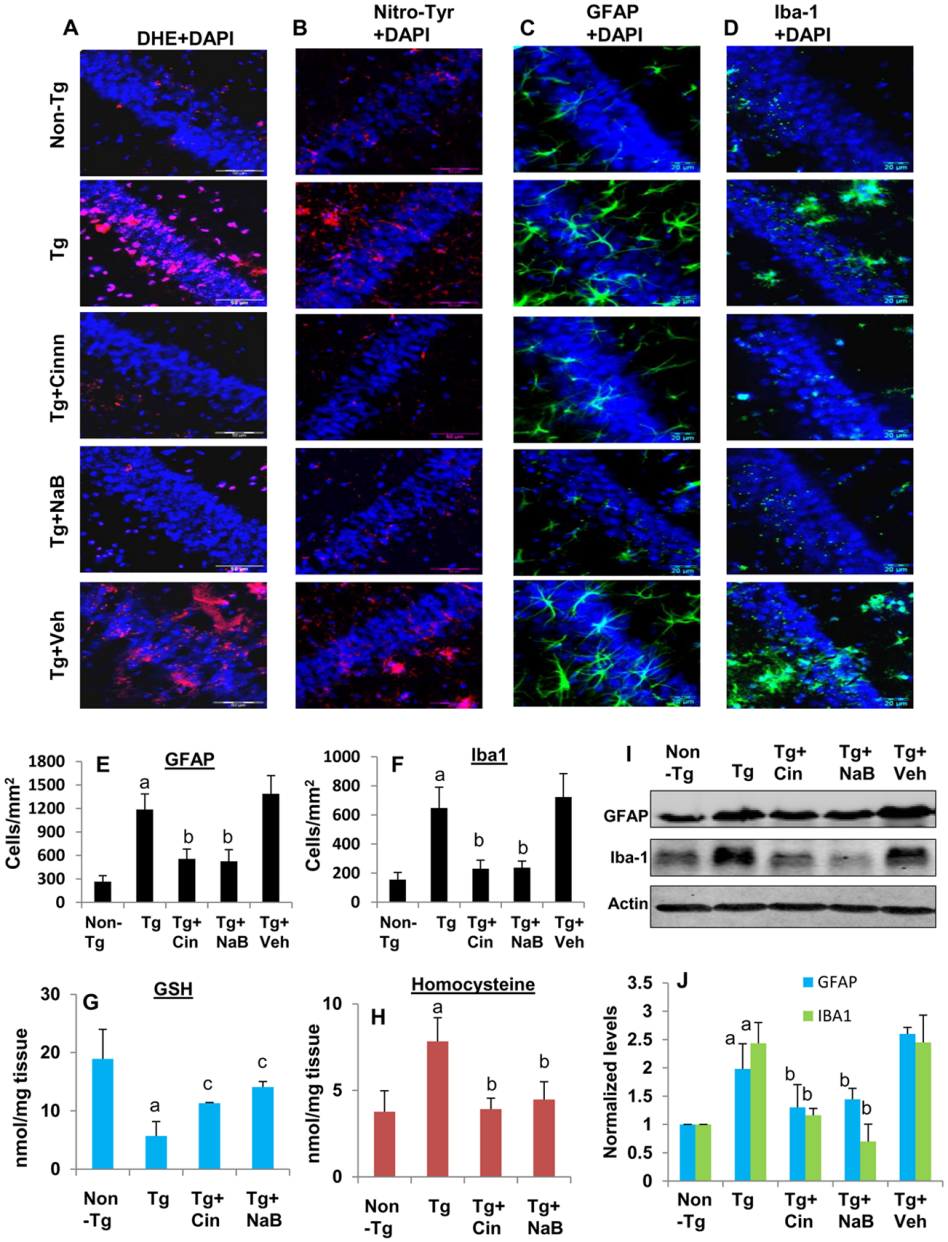
Cinnamon treatment reduces the oxidative damage and the gliosis in the hippocampus of Tg 5XFAD mice. Tg mice (6 months old) were treated with cinnamon (100 mg/kg body wt/d) and NaB (50 mg/kg body wt/d) orally. After 2 months of treatment, fresh hippocampal sections were stained for dihydroethidium (DHE) (A), nitrotyrosine (B), GFAP (C), and Iba-1 (D). Results represent analysis of two hippocampal sections of each of six mice per group. GFAP-positive (E) and Iba-1-positive (F) cells were counted in two sections (two images per slide) of each of six different brains per group. ^a^p < 0.001 vs non-Tg; ^b^p < 0.001 vs Tg. Levels of homocysteine (G) and GSH (H) were measured in the hippocampus by HPLC. Results represent mean ± SEM of six mice per group. ^*a*^ *p < 0*.*001 vs non-Tg;* ^*b*^ *p < 0*.*001 vs Tg;* ^*c*^ *p < 0*.*05 vs Tg*. I) Hippocampal homogenates were immunoblotted with antibodies against GFAP and Iba-1. Actin was run as house-keeping control. J) Bands were scanned and levels of GFAP/Actin and Iba-1/Actin were presented as relative to control. Results represent mean ± SEM of four mice per group. ^*a*^ *p < 0*.*001 vs non-Tg;* ^*b*^ *p < 0*.*01 vs Tg*.

Next, we examined if cinnamon and NaB could reduce oxidative stress in the hippocampus of Tg mice. Because NaB inhibits the production of superoxide radicals (Fig 2) and dihydroethidium (DHE) is capable of detecting superoxide radicals, hippocampal sections were stained with DHE. As expected, marked DHE staining was observed in the hippocampus of Tg mice (Fig 6A). However, treatment of Tg mice with both cinnamon and NaB decreased DHE staining (Fig 6A). This result was specific as vehicle treatment had no effect on the level of DHE (Fig 6A). One of the indices of oxidative stress is the depletion of the antioxidant glutathione (GSH), which may occur early in the development of AD [26]. Accordingly, Tg mice had low level of GSH in the hippocampus (Fig 6G). However, treatment of Tg mice with both cinnamon and NaB led to the improvement of hippocampal GSH (Fig 6G). Homocysteinemia is intimately coupled with oxidative stress and it has been shown that increased level of homocysteine is a risk factor for AD [27]. We observed increased level of homocysteine in the hippocampus of Tg mice as compared to non-Tg mice (Fig 6H). On the other hand, cinnamon and NaB treatments decreased the level of homocysteine in the hippocampus of Tg mice (Fig 6H). Superoxide is known to react with nitric oxide to produce peroxynitrite, which leaves its toxic imprint as nitrotyrosine. Accordingly, we noticed increased level of nitrotyrosine in the hippocampus of Tg mice, which was normalized by oral treatment with cinnamon and NaB (Fig 6B).

While in one hand, ROS is involved in the activation of glial cells [11], on the other, glial activation also contributes to increased oxidative stress [28]. Therefore, we also monitored gliosis in the hippocampus of treated and untreated Tg mice by immunofluorescence analysis. Increased CNS expression of Iba1, a microglia/macrophage-specific calcium-binding protein, represents microglial activation during neurodegenerative inflammation. Similarly, on activation, astrocytes also express enhanced level of GFAP, which is considered as a marker protein for astrogliosis. Levels of GFAP (Fig 6C and 6E) and Iba-1 (Fig 6D and 6F) were higher in the hippocampus of Tg mice as compared to non-Tg mice. However, cinnamon and NaB treatments reduced the expression of both GFAP (Fig 6C and 6E) and Iba-1 (Fig 6D and 6F) in the hippocampus of Tg mice. Similarly, Western blot analysis of hippocampal tissues also showed that the expression of GFAP and Iba-1protein was higher in Tg mice than non-Tg mice and that treatment of Tg mice with cinnamon and NaB led to the suppression of these glial markers (Fig 6I and 6J).

### Oral administration of cinnamon and NaB suppresses neuronal degeneration and tau phosphorylation in vivo in the hippocampus of 5XFAD Tg mice

**Fig 7:**
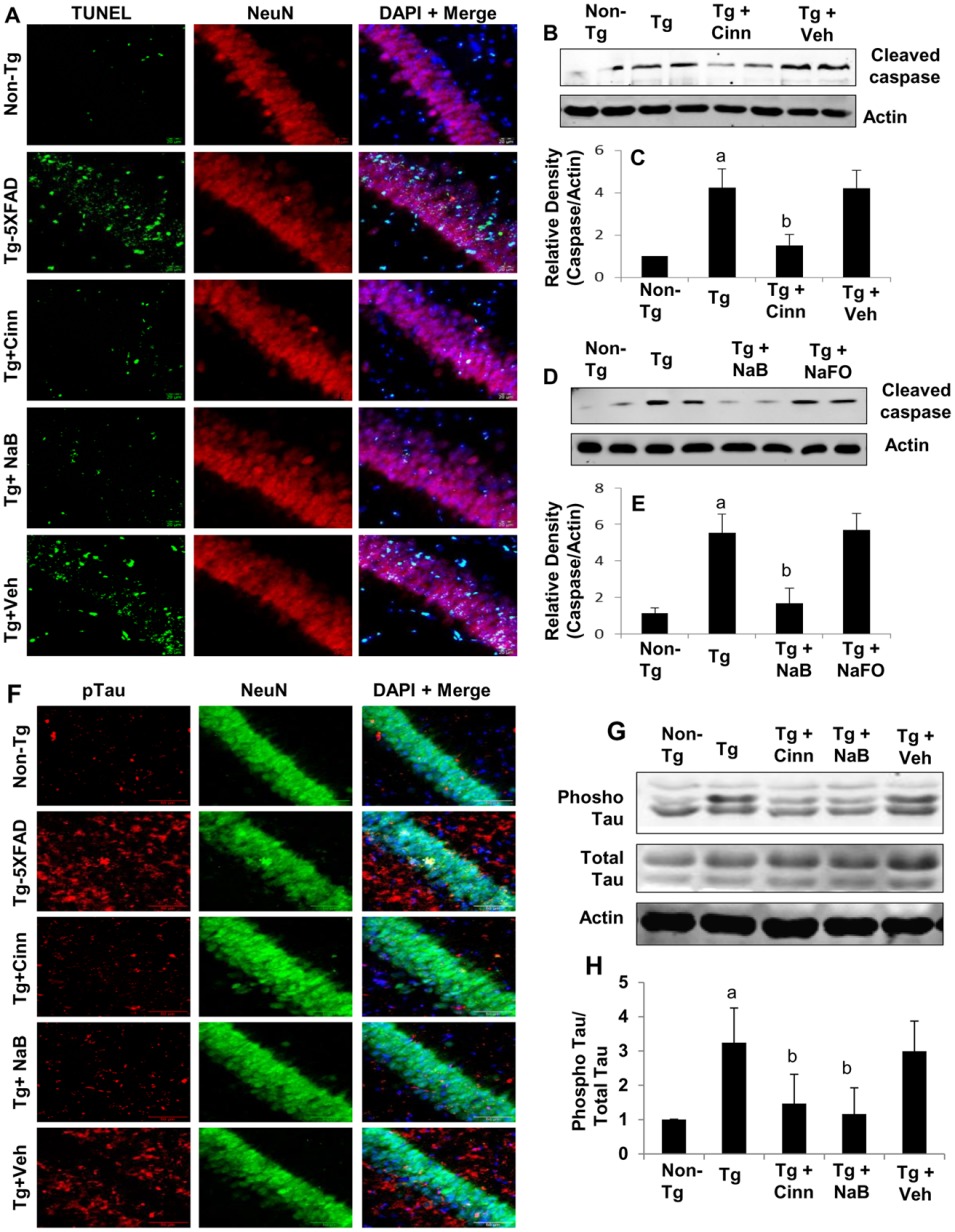
Oral administration of cinnamon powder and NaB inhibits neuronal apoptosis *in vivo* in the hippocampus of Tg5XFAD mice. Tg mice (6 months old) were treated with cinnamon (100 mg/kg body wt/d) and NaB (50 mg/kg body wt/d) orally. After 2 months of treatment, hippocampal sections were double-labeled for TUNEL and NeuN. Results represent analysis of two hippocampal sections of each of six mice per group. Tissue lysates were analyzed for cleaved caspase 3 by Western blot (B & C, cinnamon; D & E, NaB). Bands were scanned and results presented as relative to non-Tg (C, cinnamon; E, NaB). Results represent mean ± SEM of four mice per group. ^*a*^ *p < 0*.*001 vs non-Tg;* ^*b*^ *p < 0*.*001 vs Tg*. F) Hippocampal sections were double-labeled for phospho-Tau and NeuN. Results represent analysis of two hippocampal sections of each of six mice per group. G) Tissue lysates were analyzed for phospho-Tau and total Tau by Western blot. H) Bands were scanned and results presented as relative to non-Tg. Results represent mean ± SEM of four mice per group. ^*a*^ *p < 0*.*001 vs non-Tg;* ^*b*^ *p < 0*.*05 vs Tg*.

Postmortem AD brains exhibit increased neuronal TUNEL staining, suggesting that AD neurons undergo apoptosis [29] and that its reversal may have beneficial effects in AD. Therefore, we tested the effect of oral administration of cinnamon and NaB on neuronal apoptosis in the hippocampus of 5XFAD Tg mice. After 2 months of treatment, neuronal apoptosis was detected by double-labeling of hippocampal sections for NeuN and TUNEL. As expected, a number of TUNEL-positive bodies co-localized with NeuN in the CA1 region of the hippocampus of Tg mice as compared to age-matched non-Tg mice (Fig 7A). However, treatment of Tg mice with cinnamon and NaB led to the suppression of neuronal apoptosis in the hippocampus (Fig 7A). Similarly, we also detected increased level of cleaved caspase 3 in the hippocampus of Tg mice as compared to non-Tg mice (Fig 7B–7E). Oral administration of cinnamon (Fig 7B and 7C) and NaB (Fig 7D and 7E) attenuated the level of cleaved caspase 3 in the hippocampus.

Oxidative stress is known to mediate the hyperphosphorylation of tau in both cultured neurons and *in vivo* in the brain [30]. Since cinnamon and NaB decreased oxidative stress in the hippocampus of Tg mice, we examined the status of tau phosphorylation. Double labeling of hippocampal sections with NeuN and phospho-Tau showed that the level of phosphorylated tau was much higher in Tg mice as compared to age-matched non-Tg mice (Fig 7F). In contrast, oral administration of cinnamon and its metabolite NaB, but not vehicle, reduced tau phosphorylation in the hippocampus (Fig 7F). This finding was confirmed by the Western blot, where treatment with cinnamon and NaB to Tg mice lowered phospho-tau levels in the hippocampus without affecting the protein levels of total tau (Fig 7G and 7H). These results demonstrate that cinnamon and its metabolite NaB are capable of decreasing both neuronal apoptosis and tau phosphorylation *in vivo* in the hippocampus of Tg mice.

### Oral administration of cinnamon and NaB reduces plaque formation in the hippocampus of 5XFAD Tg mice

**Fig 8:**
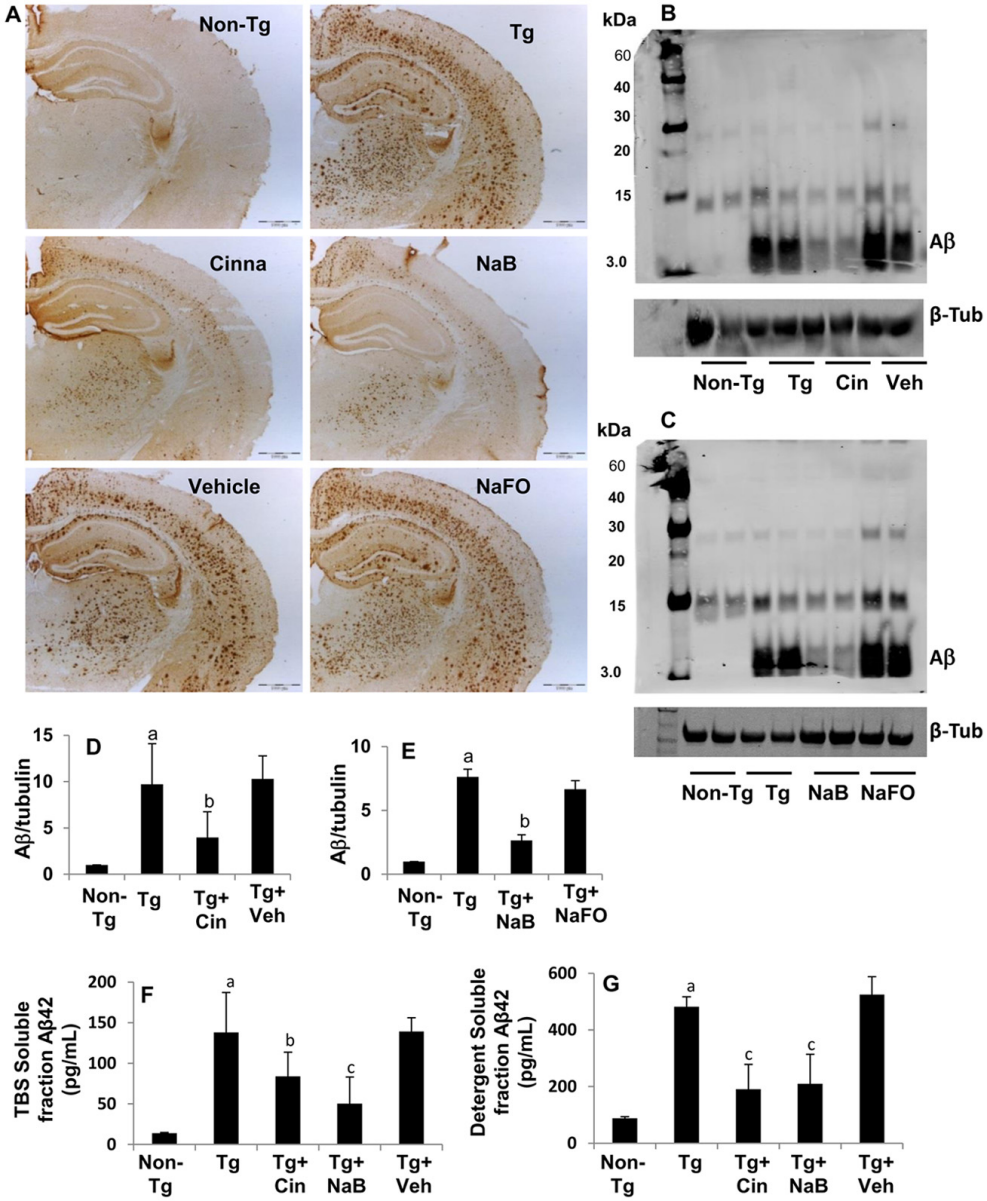
Cinnamon treatment reduces the burden of amyloid beta from the hippocampus of Tg 5XFAD mice. Tg mice (6 months old) were treated with cinnamon (100 mg/kg body wt/d) and NaB (50 mg/kg body wt/d) orally. After 2 months of treatment, hippocampal sections were immunolabeled with 82E1 antibody (A). Hippocampal homogenates were also analyzed for protein levels of Aβ by Western blot using 6E10 antibody (B, cinnamon; C, NaB). Bands were scanned and results presented as relative to non-Tg control (D, cinnamon; E, NaB). Results represent mean ± SEM of six mice per group. ^*a*^ *p < 0*.*001 vs non-Tg;* ^*b*^ *p < 0*.*01 vs Tg*. ELISA quantification of Aβ_1–42_ was performed in hippocampal homogenates (F, TBS soluble fraction; G, detergent soluble fraction). Results represent mean ± SEM of three mice per group. ^*a*^ *p < 0*.*001 vs non-Tg;* ^*b*^ *p < 0*.*05 vs Tg;* ^*c*^ *p < 0*.*01 vs Tg*.

Amyloid plaques found in the brain of AD patients are rich in Aβ peptides, which are formed after sequential cleavage of the amyloid precursor protein (APP) by α-, β- and γ-secretases. The γ secretase that produces the C-terminal end of the Aβ peptide, cleaves within the transmembrane domain of APP, generating a number of isoforms of 36–43 amino acid residues in length [31]. The most common isoforms are Aβ40 and Aβ42, which are recognized by the 82E1 and 6E10 monoclonal antibodies (mAb). We examined if cinnamon and NaB were capable of reducing the load of Aβ in the hippocampus of Tg mice. Immunostaining of hippocampal sections with 82E1 mAb (Fig 8A), immunoblot analysis of hippocampal homogenates with 6E10 mAb (Fig 8B–8E) and Aβ42 ELISA of TBS-soluble (Fig 8F) and detergent-soluble (8G) hippocampal extracts demonstrated that the level of Aβ peptides was markedly higher in the hippocampus of Tg mice as compared to non-Tg mice. However, oral treatment of Tg mice with cinnamon (Fig 8B, 8D, 8F and 8G) and NaB (Fig 8C, 8E, 8F and 8G) led to significant decrease in Aβ, indicating that cinnamon and NaB are capable of reducing the burden of Aβ in the hippocampus of Tg mice.

### Oral administration of cinnamon and NaB protects spatial learning and memory in 5XFAD Tg mice

**Fig 9:**
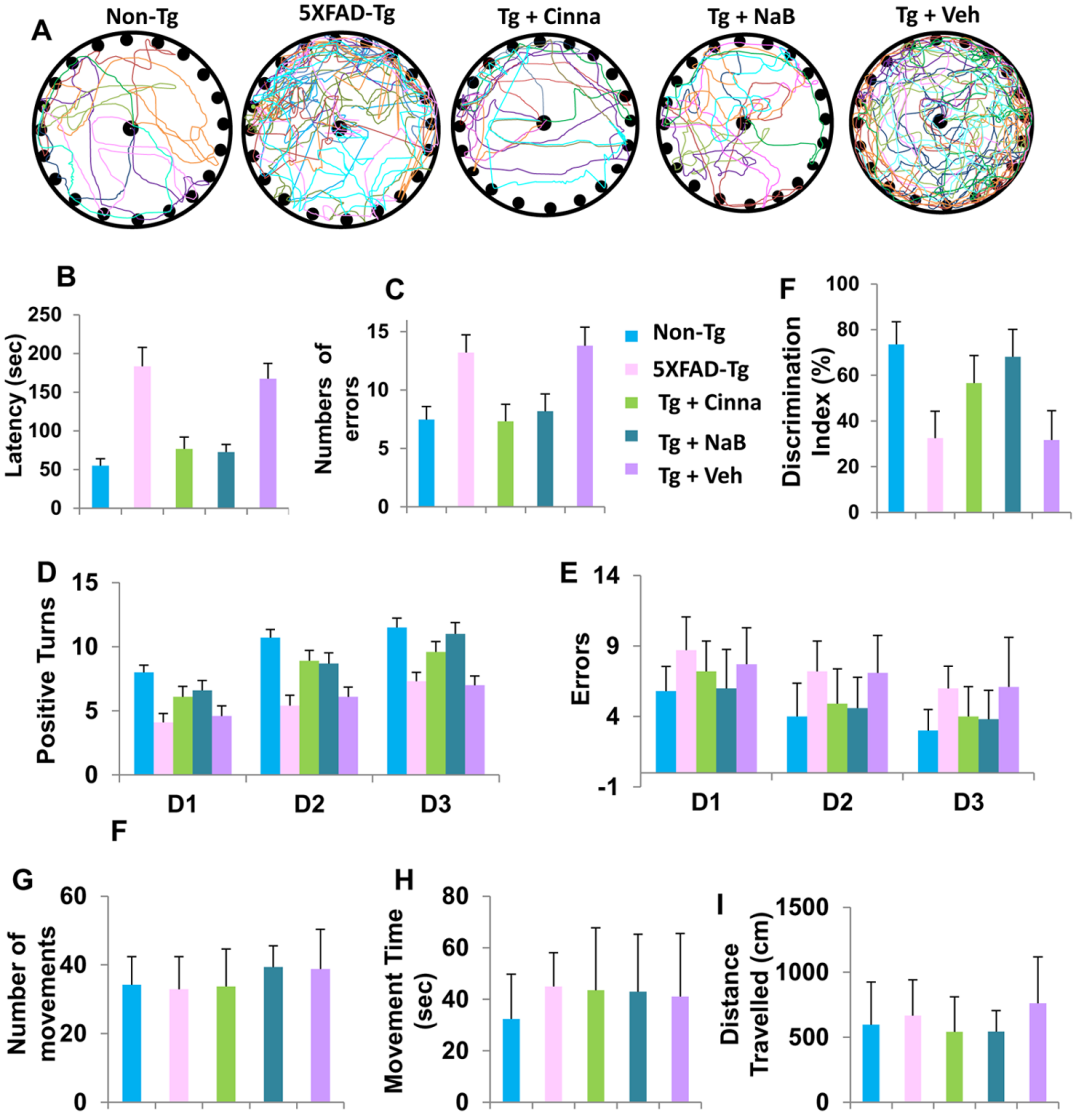
Oral administration of cinnamon powder and NaB improves memory and learning in 5XFAD Tg mice. Tg mice (6 months old) were treated with cinnamon (100 mg/kg body wt/d) and NaB (50 mg/kg body wt/d) orally. After 2 months of treatment, different groups of mice were tested for Barnes maze (A, track plot; B, latency; C, number of errors), and T maze (D, positive turns; E, errors). Short-term memory was monitored by novel object recognition test, which is represented by discrimination index (F). General locomotor activities (G, number of movements; H, movement time; I, distance travelled). Ten mice (n = 10) were used in each group.

The major problem in AD is the loss of memory and learning and the hippocampus regulates the generation of long term memory and spatial learning. Since cinnamon and NaB decreased oxidative stress, protected neurons and lowered Aβ load in the hippocampus, we examined whether oral administration of cinnamon and NaB protected only against structural damage in the hippocampus or also against hippocampus-based functional deficits seen in the Tg mice. Therefore, we evaluated Barnes maze and T maze activities. Barnes circular maze test, a hippocampus-dependent cognitive task, requires spatial reference memory. Interestingly, we found that either cinnamon-treated mice or NaB-treated mice exhibited significantly improved memory performance on Barnes maze test as shown by track plot (Fig 9A), latency [F4, 70 = 12.54, p<0.0001] (Fig 9B) and number of errors [F4, 70 = 4.972, p<0.05 (= 0.001)] (Fig 9C). *Post hoc* tests of multiple comparisons using Games-Howell analyses for latency and Tukey HSD analyses for error on Barnes maze showed that Tg mice took longer time to find the reward hole and exhibited more latency [p<0.05 (= 0.001)] and higher errors [p<0.05 (= 0.049)] in Barnes maze as compared to non-Tg mice. However, cinnamon-treated Tg mice were as efficient as healthy non-Tg in finding the target hole (Fig 9A) and exhibited significantly less latency [p<0.05 (= 0.01)] and fewer errors (p<0.05 (= 0.041)] compared to untreated Tg or vehicle treated Tg mice (Fig 9B and 9C). Similarly, NaB-treated Tg mice were as skilled as healthy non-Tg mice in finding the reward hole with less latency [p<0.05 (= 0.004)] compared to vehicle-treated Tg mice (Fig 9B and 9C).

Next, we performed T maze tests to determine cinnamon and NaB improved spatial memory in Tg mice. As expected, untreated Tg mice exhibited less number of positive turns (p<0.001) and greater number of negative turns (p<0.001) than age-matched non-Tg mice in T maze apparatus (Fig 9D and 9E). However, similar to that observed with Barnes maze, either cinnamon or NaB displayed significant effect on successful positive turns [F1, 3 = 10.345, p<0.005] (Fig 9D) and number of errors [F1, 3 = 8.417, p<0.005] (Fig 9E) as evident by one-way ANOVA repeated-measures with Tukey HSD post-hoc test.

We also monitored discrimination index, a measure for short-term memory, by novel object recognition (NOR) task, among different groups. The NOR task in particularly attractive as it requires no external motivation, reward, or punishment, and it can be completed in a relatively short time with minimal strain. In this case as well, 5XFAD Tg mice showed profound impairment [F4, 45 = 17.855, p<0.001] in short-term memory as compared to age-matched non-Tg mice (Fig 9F). Moreover, there was notable improvement [p<0.05 (= 0.018)] or [p<0.05 (= 0.012)] in short-term memory in cinnamon-treated Tg mice as compared to untreated or Vehicle-treated mice (Fig 9F). Likewise, we observed very similar NOR performance in NaB-treated Tg mice (p<0.001) as compared to Vehicle-treated mice (Fig 9F). However, either cinnamon or NaB did not significantly alter number of movements (Fig 9G), movement time (Fig 9H) and total distance travelled (Fig 9I) in Tg mice, suggesting that cinnamon and NaB do not modulate gross motor activities in this model.

## Discussion

Long-lasting and unresolved oxidative stress has long been implicated in the pathogenesis of AD. Accordingly, elevated levels of oxidative stress, including protein oxidation, lipid peroxidation and protein nitration, are histological hallmarks in either AD brain samples or in experimental models of AD [32,33,34,35,36]. Therefore, it is important to identify a safe, effective, economical, and BBB-permeable antioxidant for AD. Cinnamon, the brown bark of cinnamon tree, is a commonly used spice and flavoring material for deserts, candies, chocolate, etc. It has a long history as a medicine as well. Medieval physicians used it to treat arthritis, coughing, hoarseness, sore throats, etc. In fact, it was once so valuable, wars were fought over it. The major compound in cinnamon is cinnamaldehyde, which is converted into cinnamic acid by oxidation. In the liver, this cinnamic acid is β-oxidized to benzoyl-CoA [37] that exists as sodium benzoate (NaB). NaB is a physiological compound which is known to be excreted in the urine of human [38,39]. NaB is of medical importance as it is a component of Ucephan, a FDA-approved drug used in the treatment for hepatic metabolic defects associated with hyperammonemia such as urea cycle disorder in children [40,41]. It is also widely used as a preservative in broad range of foods and cosmetic products [42]. Earlier we have delineated that NaB modifies T cells at multiple steps and protects experimental allergic encephalomyelitis, an animal model of MS [43]. Here we demonstrated that oral administration of cinnamon powder increased the level of NaB in the hippocampus of 5XFAD mice. Interestingly, NaB exhibited antioxidant effect by suppressing the production of ROS from microglia in response to various proinflammatory stimuli.

The signaling events required for the production of ROS are becoming clear. Although there are many ROS-producing molecules, recent studies identify NADPH oxidase as the most important ROS producer in response to various inflammatory and degenerative stimuli. NADPH oxidase is a five-subunit protein that generates superoxide from molecular oxygen and is composed of two membrane-bound subunits, gp91^*phox*^ and p22^*phox*^, and at least two cytosolic subunits, p47^*phox*^ and p67^*phox*^. During its activation, p21^*rac*^ comes into the picture, associates with p67^*phox*^ and gp91^*phox*^ and completes the formation of the active enzyme complex [28]. Then this active NADPH oxidase catalyzes the formation of superoxide from molecular oxygen. Here, we observed that mevalonate and geranylgeranyl pyrophosphate (GGPP), but not farnesyl pyrophosphate (FPP), reversed the inhibitory effect of NaB on microglial superoxide production, suggesting that geranylgeranylation, but not farnesylation, is involved in the antioxidant effect of NaB. This is further supported by our results that geranylgeranyl transferase inhibitor (GGTI) inhibited the production of superoxide from activated microglia. Because GGTI inhibits geranylgeranylation of p21^*rac*^, these results suggest that p21^*rac*^ may be involved in LPS-induced microglial production of superoxide. Accordingly, NaB inhibited the activation of p21^*rac*^ in LPS-stimulated microglial cells. Similarly, oral administration of cinnamon and NaB suppressed the activation of p21^*rac*^ and increased the level of GSH *in vivo* in the hippocampus of 5XFAD mice. Since p21^*rac*^ requires geranylgeranylation for membrane attachment and activation, our results suggest that NaB suppresses geranylgeranylation of p21^*rac*^ and thereby inhibits its assembly with the NADPH oxidase complex.

There is a considerable body of evidence to suggest the involvement of oxidative stress in gliosis, plaque formation, neurofibrillary tangle formation, and neuronal death in AD. Homocysteine has pro-oxidant activity and studies have shown that increased level of homocysteine stimulates the risk of AD [27]. Consistent to the antioxidant activity, oral administration of cinnamon and NaB decreased the level of homocysteine, reduced glial activation, protected neurons from apoptosis, suppressed tau phosphorylation, and attenuated the burden of Aβ *in vivo* in the hippocampus of 5XFAD mice. The ultimate therapeutic goal of neuroprotection in AD is to improve and/or protect memory and most importantly, both cinnamon and NaB ameliorated memory impairments in 5XFAD mice. We did not notice any side effect (e.g. hair loss, weight loss, untoward infection etc.) in any of the mice used during the course of the treatment with either cinnamon or NaB. These results suggest that cinnamon and its metabolite NaB may be considered to increase hippocampal resilience and protect memory in AD.

At present, no effective therapy is available to halt the progression of AD. Administration of different inhibitors of cholinesterase such as Aricept, Exelon, Razadyne, Cognex etc has been the standard treatment for AD [44]. However, it is often associated with a number of side effects and unsatisfactory outcomes. There are several advantages of NaB and cinnamon over these proposed anti-AD therapies. *First*, both NaB and cinnamon are fairly nontoxic. Cinnamon has been widely used as flavoring material and spice throughout the world for centuries. Cinnamon is metabolized to NaB. NaB is excreted through the urine, if in excess. Furthermore, NaB is an FDA-approved drug against Urea cycle disorders in children. *Second*, cinnamon and NaB can be taken orally, the least painful route. Recently, we have demonstrated that cinnamon and NaB treatment protect mice from relapsing-remitting EAE, an animal model of MS, via regulatory T cells [10,43]. Oral administration of cinnamon and NaB also exhibits neuroprotective effect in MPTP mouse model of Parkinson’s disease [45]. One recent study has shown improvement in cognitive behavior by cinnamon extract in a mouse model of AD [46]. *Third*, cinnamon and NaB are very economical compared to other existing anti-AD therapies. *Fourth*, after oral administration, NaB rapidly diffuses through the BBB. Similarly, after oral administration of cinnamon, we also detected NaB in the hippocampus. It is thus important to see that Lin et al [47] have recently delineated improvement in cognitive and overall functions in patients with early-phase AD by NaB.

In summary, we have demonstrated that oral administration of cinnamon powder produces NaB in the hippocampus and protects memory and learning in an animal model of AD by attenuating hippocampal oxidative stress via suppression of p21^*rac*^, protecting hippocampal neurons, suppressing Tau phosphorylation, and reducing Aβ load. These results highlight a novel antioxidant role of cinnamon and its metabolite NaB and suggest that this widely-used spice and/or NaB may be explored for therapeutic intervention in AD.
